# Pulmonary Artery Banding is Still a Valuable Option

**DOI:** 10.3389/fped.2013.00017

**Published:** 2013-08-02

**Authors:** Yves Durandy

**Affiliations:** ^1^Perfusion and Intensive Care, CCML, Le Plessis-Robinson, France

In this work, Corno (1) presents long-term outcomes in a small group of patients with multiple ventricular septal defects treated with adjustable pulmonary artery banding (PAB). The results were satisfactory, with few complications. Despite recent progress in pediatric cardiac surgery, surgical management of very small babies with true Swiss cheese septum and cardiac failure is still challenging and PAB remains an option.

Optimizing classical PAB is very much a matter of experience and a number of “tricks” were developed to avoid the following two major issues:

1- Too tight or too loose? How to choose the optimal tightness of the pulmonary artery trunk in an anesthetized and mechanically ventilated patient with open chest? Three measurements can help – pressure gradient between proximal and distal pulmonary artery, balance between systemic pressure and distal pulmonary artery, and pulse oximetry – but none is fully satisfactory. The formula suggested by Trusler and Mustard (2) may be of some help but is only a guideline.
2- How to prevent distal migration in the area of pulmonary bifurcation, or even intra-lumen migration of the pulmonary artery band? How to prevent fibrotic pulmonary stenosis, subaortic stenosis, or valve damage requiring repair?

FlowWatch^®^ telemetrically adjustable PAB is likely to address both issues.

The initial intraoperative pulmonary tightness may be easily modified and adapted to the awake patient physiology and the long-term tolerance of the device seems excellent with very little pulmonary artery damage.

This sophisticated palliative surgery, leading to closure of at least some muscular ventricular septal defects and allowing patient weight gain, could be a valuable option. It could also benefit patients with significant pulmonary edema who develop critical arterial oxygen desaturation with PAB but will tolerate increasing band tightness as the pulmonary process resolves.

In this group of patients, the current approach in specialized centers is to perform complete repair rather than PAB. However, is this approach feasible everywhere and could the interesting results observed in these centers be universally reproduced?

A large randomized study is likely to be utopian and probably unethical, the choice of the best option being left to the surgeon's discretion. This choice depends on several factors, patient selection, and medical facilities being extremely different from one center to the other. Evidence-based medicine is not always required for medical progress and experience-based medicine, including human factors, is beyond question a significant contribution to improvement in patient outcome.
